# RETRACTION: “Rare tapeworm segments case report and review of literature” by Ahmed Ali Gaffer, *Clin Case Rep*. 2023;11:e7167 (https://doi.org/10.1002/ccr3.7167)

**DOI:** 10.1002/ccr3.8525

**Published:** 2024-02-17

## Abstract

The above article, published online on March 29, 2023 in Wiley Online Library (wileyonlinelibrary.com) has been retracted by agreement between the journal's Editor‐in‐Chief, Dr Charles Young, and John Wiley & Sons Ltd. The retraction has been agreed following an investigation into concerns raised by a third party that the article contains several fundamental scientific flaws. The manuscript was evaluated by a senior editor and by an independent scientific expert who both confirmed that important information contained within the manuscript and its conclusions was inaccurate.

The above article, published online on March 29, 2023 in Wiley Online Library (wileyonlinelibrary.com) has been retracted by agreement between the journal's Editor‐in‐Chief, Dr Charles Young, and John Wiley & Sons Ltd. The retraction has been agreed following an investigation into concerns raised by a third party that the article contains several fundamental scientific flaws. The manuscript was evaluated by a senior editor and by an independent scientific expert who both confirmed that important information contained within the manuscript and its conclusions was inaccurate.

